# Denitrification of Permeable Sand Sediment in a Headwater River Is Mainly Influenced by Water Chemistry, Rather Than Sediment Particle Size and Heterogeneity

**DOI:** 10.3390/microorganisms9112202

**Published:** 2021-10-22

**Authors:** Weibo Wang, Xu Wang, Xiao Shu, Baoru Wang, Hongran Li, Quanfa Zhang

**Affiliations:** 1 CAS Key Laboratory of Aquatic Botany and Watershed Ecology, Wuhan Botanical Garden, Chinese Academy of Sciences, Wuhan 430074, China; shuxiao198259420@sina.com (X.S.); lihongran@wbgcas.cn (H.L.); 2 Center of Plant Ecology, Core Botanical Gardens, Chinese Academy of Sciences, Wuhan 430074, China; 3 College of Science, Tibet University, Lhasa 850000, China; wangxu.show@outlook.com; 4 Hengyang Key Laboratory of Soil Pollution Control and Remediation, Resource Environment and Safety Engineering College, University of South China, Hengyang 421001, China; 15606975025@163.com

**Keywords:** denitrification rate, denitrification gene abundance, denitrifiers, particle size and heterogeneity, permeable sand sediment, headwater river

## Abstract

Sediment particle size and heterogeneity play an important role in sediment denitrification through direct and indirect effects on, for example, the material exchange rate, environmental gradients, microbial biomass, and grazing pressure. However, these effects have mostly been observed in impermeable sediments. On the other hand, the material exchange of permeable sediments is dominated by advection instead of diffusion, with the exchange or transport rates exceeding those of diffusion by two orders of magnitude relative to impermeable sediments. The impact of permeable sediment particle size and heterogeneity on denitrification remains poorly understood, especially at the millimeter scale. Here, we conducted an in situ control experiment in which we sorted sand sediment into four homogeneous-particle-sizes treatments and four heterogeneous treatments. Each treatment was deployed, in replicate, within the riffle in three different river reaches with contrasting physicochemical characteristics. After incubating for three months, sediment denitrifier communities (nirS, nirK, nosZ), denitrification gene abundances (nirS, nirK, nosZ), and denitrification rates in all treatments were measured. We found that most of the denitrifying microbes in permeable sediments were unclassified denitrifying microbes, and particle size and heterogeneity were not significantly correlated with the functional gene abundances or denitrification rates. Water chemistry was the key controlling factor for the denitrification of permeable sediments. Water NO_3_^−^-N directly regulated the denitrification rate of permeable sediments, instead of indirectly regulating the denitrification rate of sediments by affecting the chemical characteristics of the sediments. Our study fills a knowledge gap of denitrification in permeable sediment in a headwater river and highlights that particle size and heterogeneity are less important for permeable sediment denitrification.

## 1. Introduction

River ecosystems play an important role in the global N cycle [1]. It is estimated that about 40% of the nitrogen input into rivers is permanently removed by the denitrification of rivers at a global scale [1]. The amount of organic matter and microbial biomass in river sediments is thousands or hundreds of thousands of times that of the upper water column and is one of the hotspots for denitrification [2]. River sediments can be classified according to their particle sizes, such as silt, clay, and sand [3]. Sediments can also be classified as being permeable or impermeable [4]. As the median particle size (Dg) increases, the water permeability of sediments gradually increases [5]. When sediment permeability exceeds 10^−12^ m^2^, the sediments become permeable [4]. Fine-grained silt or clay sediments are usually defined as impermeable sediments, while coarse-grained sandy sediments (Dg > 165 μm) are permeable [6].

Compared to impermeable sediments, permeable sediments have a relatively higher DO concentration, higher grazing pressure, lower organic matter concentration, lower surface-area-to-volume ratio, and thus lower microbial biomass and a lower redox gradient [7,8]. Therefore, the traditional concept is that the denitrification of permeable sediments is limited by DO inhibition and less microbial biomass, and its denitrification potential is low [7], but recent research has challenged these traditional views [9,10].

In coastal permeable sediments, Gao et al. [11] found that denitrification is not inhibited by O_2_ and the observed high denitrification rates in the presence of O_2_ might be from the adaption of denitrifiers to redox oscillations in permeable sediments. Although permeable sediments have a low organic matter concentration and low N storage capacity, the fast convective exchange of their pore water enables them to have a faster substrate turnover rate and respiration rate [12]. Hellemann et al. [13] and Bartl et al. [14] used improved sampling and detection methods for permeable sediments and found that permeable sediments and impermeable sediments have comparable denitrification rates. Rocha [15] even referred to the permeable sandy sediment as the “fast lane” of the biogeochemical cycle.

For permeable sediments, it was found that their diversity and microbial community were shaped by water chemistry rather than sediment size and heterogeneity at the millimeter scale [16]. At the centimeter and larger scales, grain or gravel size and heterogeneity can have significant effects on the metabolic rates of sediments or substrates [17,18,19]. However, it is not clear whether their metabolic rates are affected by particle size or heterogeneity at the millimeter scale.

Here, we conducted an in-situ control experiment by artificially screening sediments with different particle sizes and heterogeneity and inoculating three reaches of a headwater river. Environmental factors, denitrification microbial communities, denitrification enzyme activities, and denitrification rates were investigated and analyzed. The key control factors of the denitrification rate of permeable sandy sediments were comprehensively analyzed, and the role of particle size and heterogeneity on the regulation of sediment property, denitrification gene abundance, and denitrification rate at the millimeter scale was systematically evaluated. We hypothesized that particle size and heterogeneity may not be limiting factors for sediment denitrification metabolism.

## 2. Materials and Methods

### 2.1. Site Description

**Figure 1:**
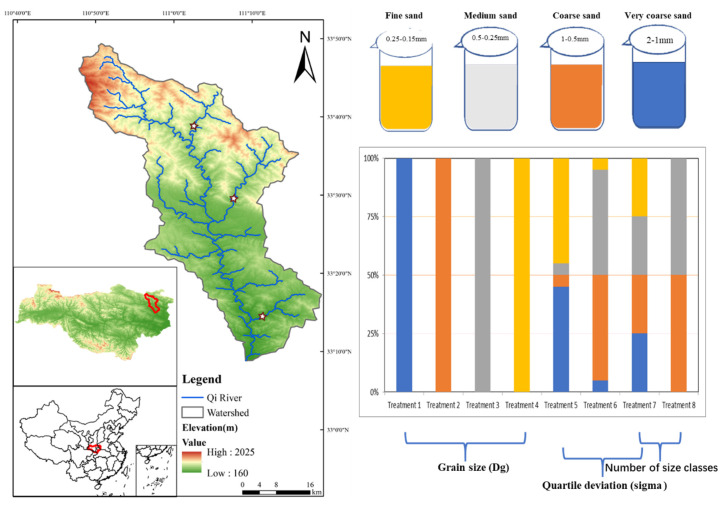
Location of the three reaches (red hollow stars) within the Qi River located on the Danjiangkou reservoir basin in central China (**left panel**) and eight treatment designs (**right panel**).

The Qi River, with a drainage area of 1501 km^2^ and a total length of 150 km, is located in the Danjiangkou Reservoir basin (Figure 1). The annual average temperature and mean precipitation of the Qi River basin were 15.1 °C and 800 mm, respectively, and over 70% of the rainfall was concentrated in June to September. The annual average TN content of the Qi River was 3.80 mg L^−1^ and ranged from 0.72 to 7.86 mg L^−1^ [16]. The main source of pollution is from non-point source pollution [20].

### 2.2. Experimental Design

Sandy sediments with different particle sizes and different heterogeneities were artificially built and inoculated in three reaches of the Qi River with different hydrological conditions and water physical and chemical characteristics (water chemistry). Four levels of grain size (Dg 1.41, 0.71, 0.35, and 0.19 mm), three levels of quartile deviation heterogeneity (sigma 2.59, 2.11, and 1.58 with the same Dg 0.50–0.52 mm), and two levels of the number of size classes heterogeneity (number 2 and 4 with the same Dg 0.50–0.51 mm) were designed (Table 1 and Figure 1). A total of eight treatments were prepared, and detailed methods were described by [16]. Briefly, river sand sediment was collected, washed, and screened according to particle size (2–1 mm, 1–0.5 mm, 0.5–0.25 mm, and 0.25–0.15 mm). The screened sand was autoclaved and mixed depending on the different design treatments (Figure 1). For each treatment in each reach, five sand packets (replicates) were set and a total of 120 packets were used. Each packet was filled with 350 mL sand and sealed. All of the packets were deployed in the riffles of the river channel on August 20 2018 and sampled for detailed analysis three months later.

### 2.3. Sediment Variables and Denitrification Rate Measurement

Total organic carbon (TOC), total nitrogen (TN), and total phosphorus (TP) of the sandy sediments in packets were measured according to standard methods [16]. The contents of sediment NH_4_^+^-N and NO_3_^−^-N were measured by extraction with a KCl solution (10 g sediment with 100 mL 2 mol KCl) and using an EasyChem Plus Discrete Analyzer (Systea, Italy). Sediment denitrification rates in the packets were measured using the acetylene (C_2_H_2_) inhibition method [21,22], the details of which have been published by Xiong et al. [21]. Briefly, fresh sediment was placed in brown glass bottles and corresponding unfiltered river water was added to the bottles. All bottles were purged with high-purity N_2_ for 2 min. C_2_H_2_ was injected into each bottle (controls without C_2_H_2_ injected) and the bottles were incubated in the dark for 2 h at 25 °C (the increase in N_2_O concentrations was linear during 2–4 h) [23]. At the start and end of sediment incubation, headspace gas was measured using a gas chromatograph (Agilent 7890, Santa Clara, CA, USA). Sediment denitrification rates were calculated as the difference between the beginning and end N_2_O concentrations divided by incubation time and sediment dry weight.

### 2.4. Denitrifier Community Analysis and Denitrification Enzyme Activity Prediction

Three denitrifier (NirS, nirK, nosZ) community compositions were identified through high-throughput amplicon sequencing, and denitrification enzyme activities were predicted by PICRUSt (Phylogenetic Investigation of Communities by Reconstruction of Unobserved States) using 16S rRNA gene data [24]. Replicate samples were pooled together, and 1 g of sand sediment was taken for DNA extraction. Primers nirS4F_nirS6R, nirK1aCuF_nirKR3CuR, nosZF_nosZ-1622R, and 338F_806R were used for gene amplification of nirS, nirK, nosZ, and 16S rRNA, respectively [16]. Amplicons were paired-end sequenced (2 × 300) on an Illumina MiSeq Sequencing platform. Raw gene sequencing reads were demultiplexed and quality filtered and OTUs were clustered with 97% similarity. The taxonomy of nirS, nirK, and nosZ OUTs was analyzed against the FunGene Database. PICRUSt was used to infer the activities (relative abundance) of nitrite reductase and nitrous oxide reductase by using the percent gene abundance, based on the 16s rRNA OUT BIOM generated from QIIME [24].

### 2.5. Denitrifier Abundance Measurement

DNA was extracted from each sand sediment sample in each packet using the PowerSoil DNA Isolation Kit (MoBio Laboratories, Inc., Carlsbad, CA, USA). The abundances (gene copies ng^−1^ DNA and gene copies g^−1^ dry sediment) of nirS, nirK, and nosZ genes were determined in triplicate using a CFX96 real-time PCR System (bio-rad, Hercules, CA, USA) with the fluorescent dye SYBR green (AceQ Master Mix, Vazyme, Nanjing, China) quantitative method. The primers that were used for amplifying the nirS, nirK, and nosZ genes are presented in Table S1. The amplification curves and dissociation curves of the qPCR are shown in Figure S1.

### 2.6. Statistical Analysis

The Shapiro–Wilk test and Bartlett test were used to assess the normality and homoscedasticity of the data, respectively. Kruskal–Wallis tests were performed to assess the significant effects of particle size, heterogeneity, and site on the sediment physicochemical properties, gene abundances, enzyme activities, and sediment denitrification rates. Wilcoxon rank sum tests were then conducted to make multiple comparisons if it was determined that there was a statistical significance. Pearson correlation analyses were used to evaluate the correlations between environmental factors and between sediment denitrification rates and environmental factors. Stepwise multiple regression analysis was used to identify the key environmental factors of sediment denitrification rates. Structural equation modeling (SEM) was used to further explore the direct and indirect effects of the biotic and abiotic environmental factors on sediment denitrification rates. The comparative fit index (CFI), the root square error of approximation (RMSEA), and the standardized root mean square residual (SRMR) were used to test the acceptability of the fit of the model. The analysis of similarity (ANOSIM) statistics was calculated to test the significance of community differences among sediments from different reaches. Statistical tests and plots were performed using the R package vegan [25,26], and customized R-scripts.

## 3. Results

### 3.1. Changes of Abiotic Environmental Factors

**Figure 2:**
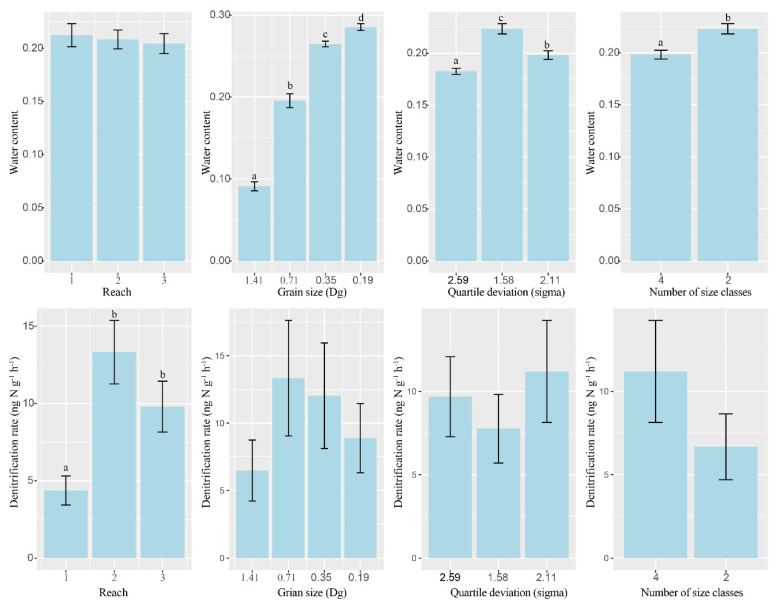
Different water contents and denitrification rates under different reaches and different particle sizes and heterogeneity. Different letters above the columns indicate significant differences (*p* < 0.05).

There were significant differences in the nutrient levels between the three river reaches, but all of the nutrient levels were lower than the average nutrient level of the river (Table S2). Significant negative correlations were found between water electrical conductivity (EC) and sediment NH_4_^+^-N and TN, while water NO_3_^−^-N and sediment NH_4_^+^-N and TN were significantly positively correlated (Figure S2). Sediment TOC and TP were positively correlated with sediment water content, but there were no significant correlations between sediment NH_4_^+^-N and TN and sediment water content (Figure S3). Sediment particle size and heterogeneity significantly changed the physical and chemical characteristics of sediment (Table 2). The water content of the sediments gradually increased as particle size decreased, decreased as quartile heterogeneity increased, and decreased as the number of heterogeneous size classes increased. However, they were not significantly different between sediments from different river reaches (Figure 2). The TOC, TN, and TP of the sediments increased significantly as particle size decreased, while NH_4_^+^-N and NO_3_^−^-N did not change as particle size changed. TN increased as quartile heterogeneity increased, and other indicators showed no significant variation with changes in sediment heterogeneity (Figure S4).

### 3.2. Three Denitrifier Community Compositions and Their Relationships with Abiotic Environmental Facotors

**Figure 3:**
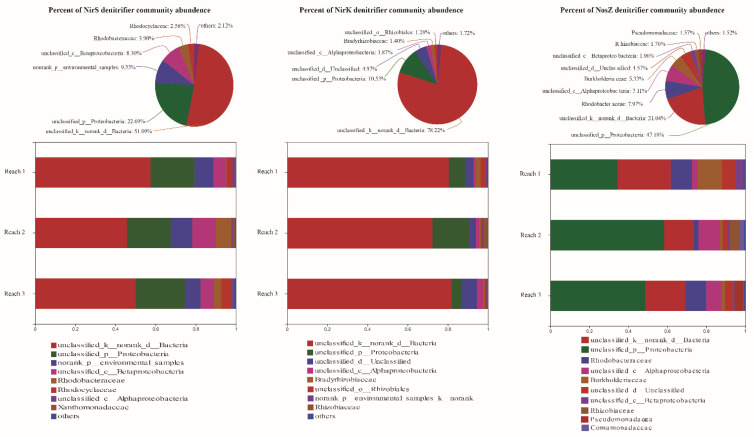
nirS, nirK, and nosZ denitrifier communities (on family level against the FunGene database) in permeable sandy sediments from three different reaches.

The community compositions of the three types of denitrifiers based on a comparison of amplicons with the FunGene database are shown in Figure 3. For three types of denitrifiers, only the dominant taxa of the NosZ denitrifier community could be affiliated to the phylum level, while the dominant taxa of the other two types of denitrifiers could not be affiliated. Some common denitrifier taxa, such as *Brucellaceae* and *Comamonadaceae*, were not detected. Some taxa could be detected, but their contents were low, such as *Rhodobacteraceae* and *Rhodocyclaceae*, which accounted for only 3.90% and 2.58% of the nirS denitrifier community, respectively. Meanwhile, some uncommon microbial taxa, such as *Bradyrhizobiaceae*, were found in the nirK community. There were significant differences for three denitrifier communities among different river reaches (ANOSIM, all *p* < 0.05), but particle size and heterogeneity did not significantly change three denitrifier microbial communities (Figure 3 and Figure S5).

### 3.3. Denitrification Gene Abundances and Their Relationships with Abiotic Environmental Factors

**Figure 4:**
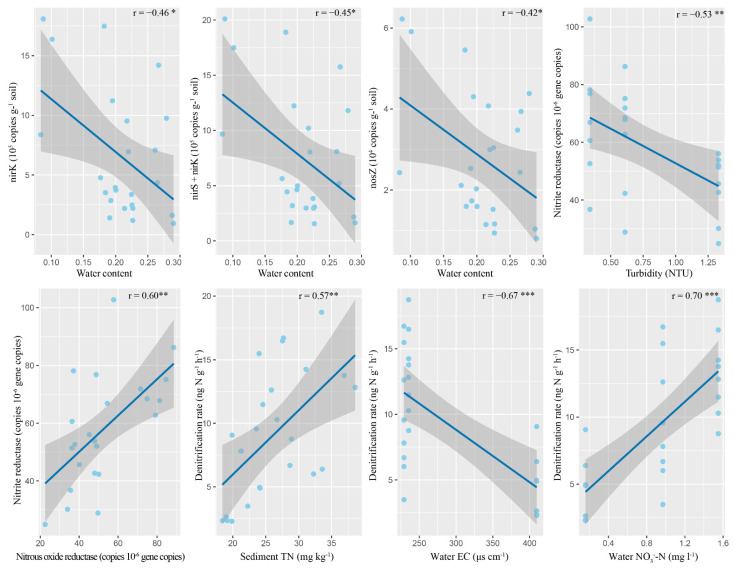
Significant Pearson correlations between denitrification rates, water parameters, sediment parameters, gene abundances, and enzyme activities. * significant level < 0.05; ** significant level < 0.01; *** significant level < 0.001.

The absolute abundances of nirK, nirS, and nosZ genes were 6.57 × 10^6^, 9.40 × 10^5^, and 2.79 × 10^5^ copies g^−1^ soil, and their coefficients of variation (CV) were 0.82, 0.51, and 0.58, respectively. The relative abundances of nirK, nirS, and nosZ genes were 2.54 × 10^5^, 3.71 × 10^4^, and 1.09 × 10^4^ copies ng^−1^ DNA, respectively. There were no significant differences in the abundance of these three denitrification genes in different river reaches. In most cases, particle size and heterogeneity could not significantly change the denitrification gene abundances, except nirS (*p* = 0.05) (Table 3). No positive correlations were found between denitrification gene abundances and other water and sediment characteristics (Table 4, Figure 4).

### 3.4. Denitrification Enzyme Activities and Their Relationships with Abiotic Environmental Factors

**Figure 5:**
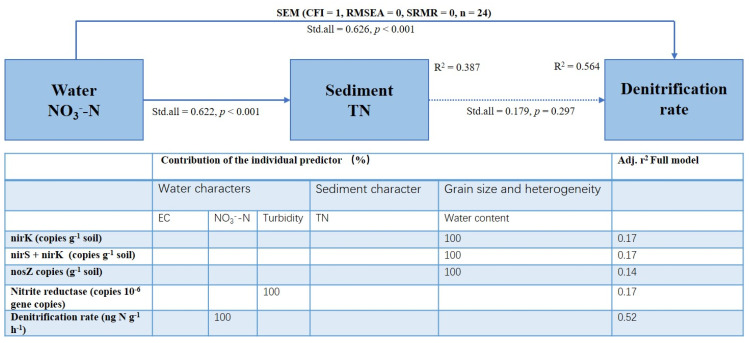
Model summary for the SEM and the stepwise multiple regression of denitrification factors on five environmental variables. Continuous and dashed arrows indicate significant and insignificant relationships.

The activities of nitrite reductase and nitrous oxide reductase were predicted by PICRUSt. The Kruskal–Wallis test showed that neither particle size nor heterogeneity could change their activities, but their activities differed significantly in different inoculated river reaches. Both appeared to be significantly lower in Reach 3 than in Reaches 1 and 2 (Table 3). The activities of the two enzymes were significantly positively correlated (Figure 4), but they were not significantly positively correlated with the physical and chemical characteristics of the sediments (Table 4). Nitrite reductase activity was negatively correlated with water turbidity (Table 4, Figure 4). The stepwise regression analysis showed that turbidity could explain the variation in nitrite reductase activity (Figure 5).

### 3.5. Denitrification Rates and Their Relationships with Abiotic and Biotic Environmental Factors

The average denitrification rate of permeable sediment was 9.50 ng N g^−1^ h^−1^, and the CV was 1.14. The Kruskal–Wallis tests showed that both the particle size and heterogeneity did not change the denitrification rate of sandy sediments. However, the sediment denitrification rate was significantly different in different river reaches (Table 3). The Wilcoxon rank sum tests showed that the denitrification rate of sandy sediments in Reach 1 was significantly lower than Reach 2 and Reach 3 (Figure 2). Sediment denitrification rates and the abundance of three denitrification genes (nirS, r = −0.03, *p* = 0.89; nirK, r = 0.20, *p* = 0.35; nosZ, r = 0.11, *p* = 0.60) were not significantly correlated, and they were not significantly positively related to the two denitrification enzyme activities (Nitrite reductase, r = −0.04, *p* = 0.86; Nitrous oxide reductase, r = −0.52, *p* = 0.01). However, the denitrification rates were significantly positively correlated with water NO_3_^−^-N and sediment TN (Table 4, Figure 4). The stepwise regression analysis showed that water NO_3_^−^-N could explain all the variation of the denitrification rate (Figure 5). SEM analysis showed that the water NO_3_^−^-N directly affected the denitrification rate, rather than indirectly through the impact on the sediment TN. This model could explain the 56% variation in the denitrification rate (Figure 5).

## 4. Discussion

### 4.1. Characteristics of Denitrifier Communities and Denitrification Rates in Permeable Sandy Sediments

For the three denitrifier communities, only the dominant taxa of the NosZ denitrifier community could be affiliated, while the other two denitrifier communities could not be affiliated for their respective dominant taxa. This result indicated that there might be unknown dominant denitrifying taxa in the permeable sandy sediments.

Compared with the natural sediments in the Qi River (nirK, 4.18 × 10^5^ copies g^−1^ soil; nirS, 5.42 × 10^6^ copies g^−1^ soil; nosZ, 3.84 × 10^4^ copies g^−1^ soil, unpublished data), the nirS abundances of inoculated sandy sediments were lower than the natural sediments, but the nirK and nosZ abundances were higher than the natural sediments. We found that the nirK abundance was higher than the nirS abundance in inoculated sandy sediments. In most cases, the abundance of nirS was much greater than that of nirK in sediments. This pattern may be related to the nirK genes’ preference for an oxygen-sufficient environment [27,28].

Compared with the average denitrification rate of the entire river sediment (mean value = 45.37 ng N g^−1^ h^−1^, CV = 0.95, unpublished data), the denitrification rates of the inoculated sandy sediments were lower. This difference may be related to the nutrient level of the water. The nutrient levels of the three reaches selected in this study were significantly lower than the average nutrient level of the river (13 reaches evenly distributed throughout the river).

### 4.2. Key Factors Controlling Denitrification of Permeable Sandy Sediments

The sediment denitrification rate is comprehensively regulated by different abiotic environmental factors and biotic environmental factors [29,30], but for different habitats and substrate types, the key control factors may be different. We found that for permeable sandy sediments, although the denitrification rate was significantly related to the sediment TN, water NO_3_^−^-N was the key controlling factor. There was no significant correlation between denitrification functional gene abundance and the denitrification rate. In other words, the denitrification rate was not significantly affected by biological environmental factors or microbial functional gene attributes. However, for impermeable sediments or terrestrial soils, nutrient and functional gene abundance usually significantly affects the denitrification rate [22,31,32].

In general, we concluded that the denitrification rates of permeable sandy sediments in our study were limited by substrates, not by the catalytic enzymes. More interestingly, the denitrification rates of the permeable sandy sediments were limited by the N substrate, while in the natural muddy sediments, we found that their denitrification rates were limited by the C substrate. In fact, in most ecosystems, the rate of denitrification was more susceptible to the limitation of C substrate, and the limitation of C substrate can only be lifted when a large amount of organic matter is imported [33]. The N restriction in this study might be related to the high C/N ratio [34]. The average DOC/NO_3_^−^-N of the overall river was 1.52, and the average ratio value of the three reaches was 13.84. For impermeable sediments, the substrate limitation may come from a substrate diffusion limitation, especially for labile organic carbon [35]. In these sediments, denitrifiers face competition for C sources from non-denitrifying heterotrophic microorganisms, while the competition for inorganic nitrogen sources is much weaker. For permeable substrates, free diffusion is replaced by advection processes and the rate is increased by two orders of magnitude [4]. Advection processes may help alleviate the C limitation. On the one hand, recent studies have shown that there is a coupled process of nitrification and denitrification in oxygen-rich river sandy sediments [36]. Whether there are some microbial taxa that can complete the coupled process of nitrification and denitrification alone needs further investigation. Nitrifying microbes are autotrophic microorganisms. If some of them have both nitrification and denitrification functions [36], then the C demand for the denitrification process will be greatly reduced. Based on our inference, the denitrification process of permeable sandy sediments may be much less dependent on the C source than that of impermeable sediments.

### 4.3. Impacts of Particle Size and Heterogeneity on the Denitrification Process of Permeable Sandy Sediments

**Figure 6:**
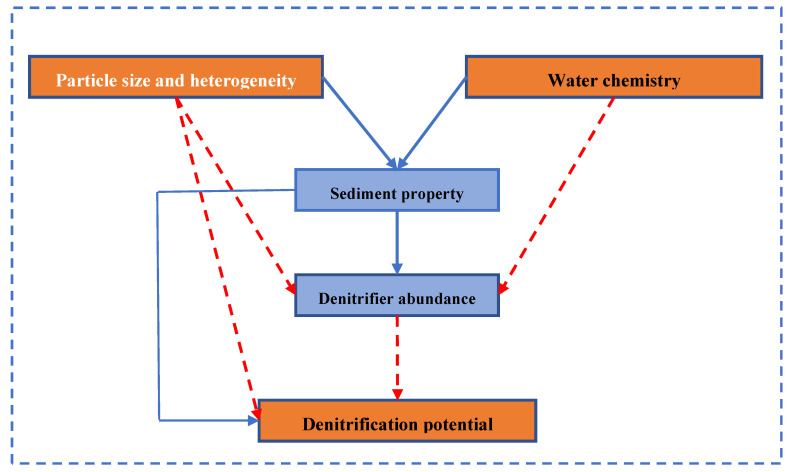
Schematic demonstrating that water chemistry, rather than particle size and heterogeneity, sediment properties, and function gene abundances, was the most important factor controlling the denitrification rates of permeable sandy sediments. Continuous and dashed arrows indicate significant and insignificant relationships. The width of arrows is proportional to the strength of relationships.

Our findings indicated that particle size and heterogeneity did change the chemical or nutritional characteristics of sandy sediments as we expected, but they did not regulate the abundances of denitrifying functional genes, nor could they regulate the expression of denitrifying enzymes. Similarly, particle size and heterogeneity did not change the denitrification rate of sandy substrates (Figure 6). Our results were different from those reported by previous studies. For example, Highton et al. [37] found that particle size significantly affected the abundance of denitrification functional genes and denitrification potential. For soils of different particle sizes, the smaller the particle size, the greater the area ratio, and the greater the microbial biomass [7,8,38]. However, cell densities on individual sand grains are more dependent on the sphericity of the grains, rather than on their size for permeable sands [39]. Hence, higher surface areas for sands did not mean higher surface area available for microbes. Besides, microbial biomass and their ecological function are not always related [40], so the higher microbial biomass did not mean a higher denitrification potential. On the other hand, we speculated that there might be a threshold for particle size and heterogeneity factors, both for freshwater and marine sediments, below which they will have an effect, and the particle size in our study was above the threshold. For example, the particle diameters in our study were all larger than 150 μm, and related studies found the correlation between particle sizes and denitrification rates only when the particle diameters were less than 100 μm [29].

### 4.4. Problems and Prospects of the Research

Here, we measured the denitrification rate using the traditional acetylene inhibition method, and the incubation method was stable culture, in order to compare the results of the natural sediments and riparian soils in the same river [32]. However, recent studies have found that the stable incubation method greatly underestimated the denitrification rate [14], because the advection exchange rate of the material in the permeable sediment was much higher than the free diffusion rate. Improved methods may be used in the future [13] and are expected to deepen our understanding of the denitrification potential of permeable sandy sediments.

Sandy sediments in the river are more open than muddy sediments. How their metabolic processes respond to the rapidly changing external environment is unknown, whether they respond quickly or remain relatively stable. Studies have shown that the nitrogen removal process of the permeable substrate was highly temporally variable [14]. Although the results of our study indicated that particle size and heterogeneity did not change the denitrification potential, whether it can change the fluctuations of the denitrification potential is worthy of further investigation. Moreover, our study was only a one-time survey, and long-term continuous monitoring needs to be conducted to better understand these relationships over time.

We found that the nirK abundance of sediments determined by qPCR in the headwater river was significantly greater than nirS abundance, which differed from the results reported by most other studies [41]. Although many studies have shown that a high DO environment supports nirK gene expression, the mechanism is still unclear. Whether they change the microbial community or change the ability of nirK gene drift remains unknown.

## 5. Conclusions

Our results indicated that both particle size and heterogeneity and water chemistry had a significant impact on permeable sediment property but had no impact on denitrification gene abundances. Water chemistry rather than particle size and heterogeneity regulated the denitrification potential. The abundance of microbial nitrite and nitrous oxide reductase genes did not regulate the denitrification rate in the permeable sediments. Denitrification in permeable sediments was limited by N sources rather than carbon sources.

## Figures and Tables

**Table 1:** Experimental treatment designs to test effects of particle size and heterogeneity on permeable sediment denitrification.

Treatment Design	Particle Size				Particle Heterogeneity				
					Quartile Deviation			Number of Size Classes	
Treatment	1	2	3	4	5	6	7	7	8
Number of size classes (nsc)	1	1	1	1	4	4	4	4	2
Geometric mean grain size (Dg) (mm)	1.41	0.71	0.35	0.19	0.52	0.50	0.51	0.51	0.50
Geometric standard deviation (sigma)	1.00	1.00	1.00	1.00	2.59	1.58	2.11	2.11	1.41
Permeability (10^−11^ m^2^)	144.21	36.57	8.89	2.62	19.61	18.13	18.87	18.87	18.13

**Table 2:** Different sediment chemical parameters under different particle sizes and heterogeneities and reaches as illustrated by the Kruskal–Wallis test. Values in bold indicate significant differences (*p* < 0.05).

Sediment Characteristics	Reach		Particle Size		Particle Heterogeneity			
					Quartile Deviation		Number of Size Classes	
	Kruskal–Wallis chi-squared	*p*-value	Kruskal–Wallis chi-squared	*p*-value	Kruskal–Wallis chi-squared	*p*-value	Kruskal–Wallis chi-squared	*p*-value
TOC (g kg^−1^dw)	1.47	0.48	**13.61**	**<0.01**	0.42	0.81	0.01	0.93
TN (mg kg^−1^dw)	**28.36**	**<0.01**	**9.04**	**0.03**	**8.31**	**0.02**	1.50	0.22
TP (mg kg^−1^dw)	**7.36**	**0.03**	**23.58**	**<0.01**	5.24	0.07	0.53	0.47
NH_4_^−^-N (mg kg^−1^fw)	**15.45**	**<0.01**	5.16	0.16	0.51	0.77	2.65	0.10
NO_3_^−^-N (mg kg^−1^fw)	**6.72**	**0.03**	2.65	0.45	0.01	0.99	0.14	0.70

**Table 3:** Different denitrification gene abundances and enzyme activities under different particle sizes and heterogeneities and reaches as illustrated by the Kruskal–Wallis test. Values in bold indicate significant differences (*p* < 0.05).

Microbial Abundance and Enzyme Activity	Reach		Particle Size		Particle Heterogeneity			
					Quartile Deviation		Number of Size Classes	
	Kruskal–Wallis chi-squared	*p*	Kruskal–Wallis chi-squared	*p*	Kruskal–Wallis chi-squared	*p*	Kruskal–Wallis chi-squared	*p*
nirS (copies ng^−1^ DNA)	3.71	0.16	2.49	0.48	0.09	0.96	**3.86**	**0.05**
nirS (copies g^−1^ soil)	1.94	0.38	4.03	0.26	0.27	0.86	**3.86**	**0.05**
nirK (copies ng^−1^ DNA)	4.12	0.13	5.16	0.16	0.27	0.88	0.43	0.51
nirK (copies g^−1^ soil)	3.85	0.15	6.59	0.09	0.62	0.73	1.19	0.28
nirS + nirK (copies ng^−1^ DNA)	2.99	0.22	5.15	0.16	0.27	0.88	1.19	0.28
nirS +nirK (copies g^−1^ soil)	3.19	0.20	6.59	0.09	0.62	0.73	1.19	0.28
nosZ (copies ng^−1^ DNA)	1.99	0.37	4.59	0.20	0.80	0.67	0.05	0.83
nosZ (copies g^−1^ soil)	2.56	0.28	5.36	0.15	2.22	0.33	0.05	0.843
Nitrite reductase (copies 10^−6^ gene copies)	**7.29**	**0.03**	3.00	0.39	1.16	0.56	0.43	0.51
Nitrous oxide reductase (copies 10^−6^ gene copies)	**11.42**	**<0.01**	1.67	0.64	1.42	0.49	0.05	0.83

**Table 4:** Pearson correlation coefficients between denitrification factors (denitrifier abundances, enzyme activities, and denitrification rates) and water and sediment characteristics. * significant level < 0.05; ** significant level < 0.01.

	Water Characteristics				Sediment Characteristics				
	EC	Turbidity	NO3-N	Water content	STOC	STN	STP	SNH_4_^+^-N	SNO_3_^−^-N
nirS (copies ng^−1^ DNA)	−0.08	0.36	−0.09	−0.07	0.05	−0.10	0.28	−0.20	0.34
nirS (copies g^−1^ soil)	−0.06	0.28	−0.06	−0.24	0.02	−0.08	0.19	−0.18	0.27
nirK (copies ng^−1^ DNA)	−0.26	0.04	0.25	−0.35	−0.22	0.02	0.10	0.01	0.03
nirK (copies g^−1^ soil)	−0.26	−0.05	0.29	−0.46 *	−0.22	0.09	<0.01	0.09	−0.04
nirS + nirK (copies ng^−1^ DNA)	−0.26	0.07	0.23	−0.33	−0.20	0.01	0.12	−0.01	0.06
nirS +nirK (copies g^−1^ soil)	−0.25	−0.03	0.27	−0.45 *	−0.21	0.08	0.02	0.07	−0.01
nosZ (copies ng^−1^ DNA)	−0.24	0.07	0.21	−0.25	−0.20	0.02	0.10	0.05	0.05
nosZ (copies g^−1^ soil)	−0.24	−0.06	0.28	−0.42 *	−0.21	0.12	−0.02	0.14	−0.03
Nitrite reductase (relative gene copies)	0.07	−0.53 **	0.14	0.19	0.39	0.09	−0.15	−0.27	−0.13
Nitrous oxide reductase (relative gene copies)	0.31	−0.37	−0.14	012	0.37	−0.09	−0.29	−0.37	−0.08
Denitrification rate (ng N g^−1^ h^−1^)	−0.67 **	−0.11	0.74 **	0.12	0.12	0.57 **	0.16	0.28	−0.13

## Supplementary Materials

The following are available online at https://www.mdpi.com/article/10.3390/microorganisms9112202/s1, Table S1: The primers information used for qPCR in this study; Table S2: Hydrodynamics and water physico-chemistry in the Qi River and the three inoculated reaches; Figure S1: The amplification and dissociation curves of the qPCR; Figure S2: Correlations between water chemistry and sediment chemical characteristics; Figure S3: Correlation matrix of sediment physical and chemical characteristics; Figure S4: The chemical parameters of the sandy sediment with different reaches, particle median sizes, and heterogeneities; Figure S5: Three denitrifier community compositions (at the family level) within 24 different treatments.
