# Supplementary material for: Denitrification of Permeable Sand Sediment in a Headwater River Is Mainly Influenced by Water Chemistry, Rather Than Sediment Particle Size and Heterogeneity
Source: Microorganisms. 2021 Oct 22;9(11):2202. doi: 10.3390/microorganisms9112202 (PMC8624688; doi:10.3390/microorganisms9112202)
Supplement: Supplementary file 1 [file microorganisms-09-02202-s001.zip › microorganisms-1379821-supplementary.pdf]

Table S1. The primers information used for qPCR in this study.

Name	Sequence (5'-3')	Types	PCR Products (bp)
**nirS**	TTCRTCAAGACSCAYCCGAA	Forward Primer	331
	CGTTGAACTTRCCGGT	Reverse Primer	
**nirK**	ATCATGGTSCTGCCGCG	Forward Primer	165
	GCCTCGATCAGRTTGTGGTT	Reverse Primer	
**nosZ**	CGYTGTTCMTCGACAGCCAG	Forward Primer	252
	CGSACCTTSTTGCCSTYGCG	Reverse Primer	

Table S2. Hydrodynamics and water physico-chemistry in the Qi River and the three inoculated reaches (Wang et al., 2021). Values in bold and different letters indicate significant differences ($p < 0.05$).

River and Reach	River	Reach 1	Reach 2	Reach 3
**Temperature** (°C)	15.06 ± 8.31	17.80 ± 7.16	14.23 ± 6.84	15.12 ± 9.03
**Pressure** (mmHg)		**736.73 ± 4.57 b**	**700.2 ± 9.04a**	**703.8 ± 7.04 a**
**Turbidity** (NTU)		**0.61 ± 0.12 ab**	**0.34 ± 0.17 a**	**1.33 ± 0.87 b**
**Water velocity** (m s^{-1})		0.23 ± 0.2	0.17 ± 0.11	0.29 ± 0.15
**EC** (μs cm^{-1})		**409.63 ± 40.92 b**	**235.48 ± 25.89 b**	**229.13 ± 14.88 a**
**pH**	8.18 ± 0.24	8.54 ± 0.14	8.50 ± 0.17	8.49 ± 0.36
**ORP** (mv)		282.2 ± 45.99	281.45 ± 43.67	283.22 ± 43.21
**TC** (mg L^{-1})		26.8 ± 8.65	24.21 ± 8.72	21.95 ± 5.99
**TIC** (mg L^{-1})		9.7 ± 12.6	8.87 ± 10.33	6.97 ± 8.03
**TOC** (mg L^{-1})		17.09 ± 4.19	15.34 ± 6.14	14.97 ± 7.02
**DOC** (mg L^{-1})	2.47 ± 1.27	13.08 ± 5.53	13.08 ± 8.35	10.90 ± 5.79
**TN** (mg L^{-1})	3.80 ± 1.45	1.66 ± 0.9	2.89 ± 0.95	2.47 ± 2.39
**NH_4^+-N** (mg L^{-1})	0.50 ± 0.34	0.12 ± 0.15	0.08 ± 0.06	0.10 ± 0.12
**NO_3^--N** (mg L^{-1})	1.63 ± 0.81	**0.16 ± 0.16 a**	**1.55 ± 0.42 b**	**0.97 ± 0.50 ab**
**TP** (mg L^{-1})	0.03 ± 0.02	0.02 ± 0.01	0.02 ± 0.01	0.03 ± 0.02
**DO** (mg L^{-1})	9.48 ± 1.59	11.42 ± 3.83	10.18 ± 2.64	11.12 ± 1.96

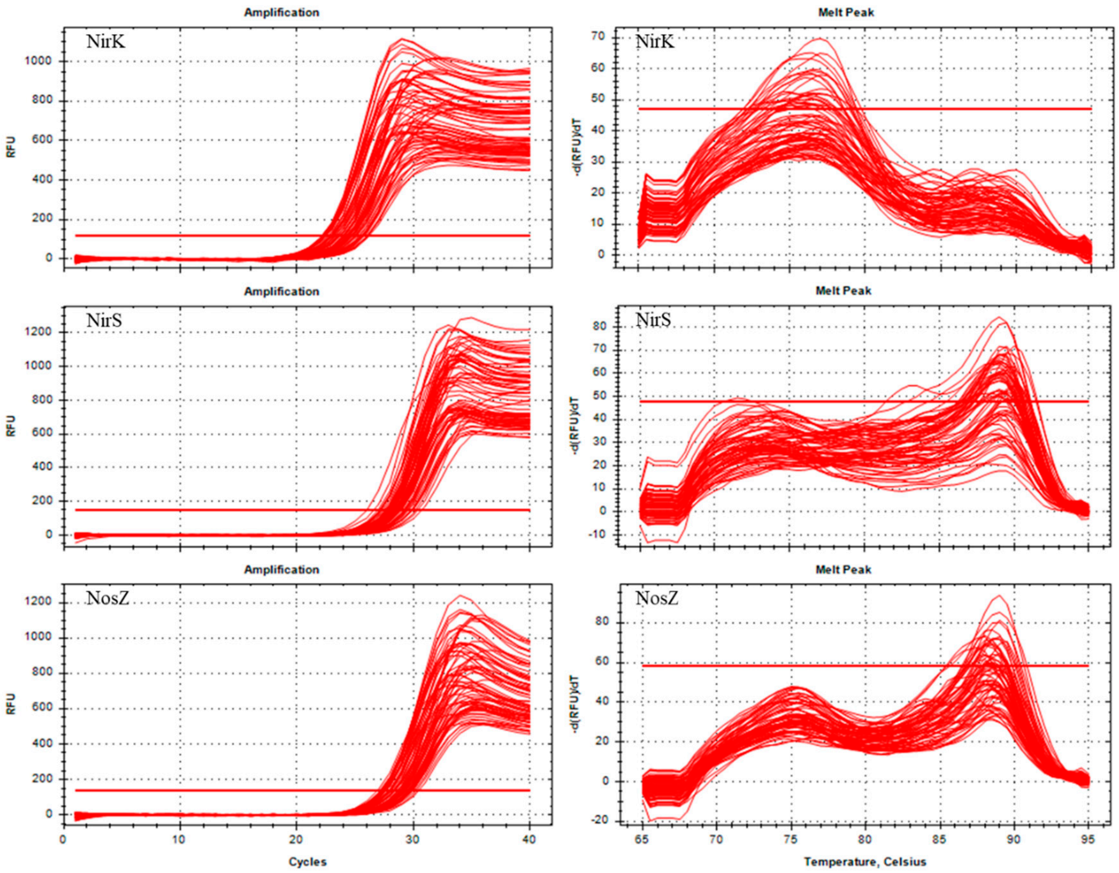

Figure S1. The amplification and dissociation curves of the qPCR.

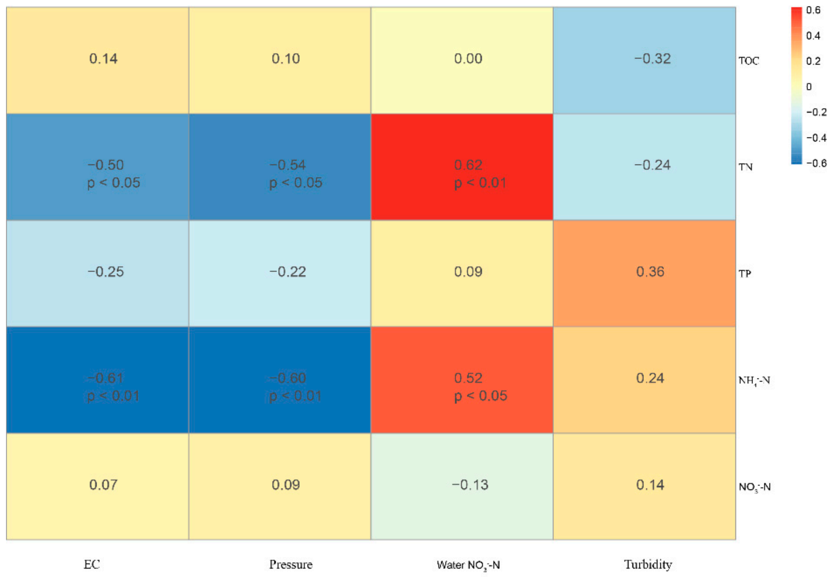

Figure S2. Correlations between water chemistry and sediment chemical characteristics.

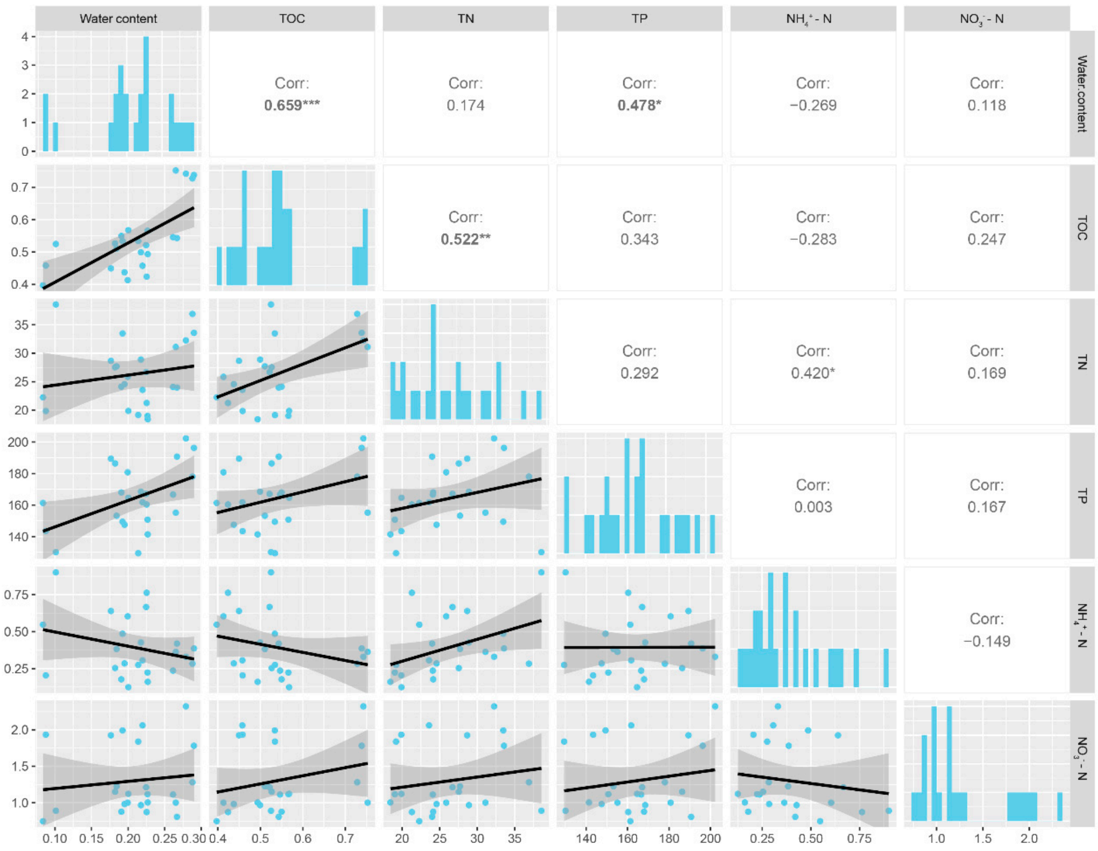

Figure S3. Correlation matrix of sediment physical and chemical characteristics. * significant level < 0.05; ** significant level < 0.01; *** significant level < 0.001.

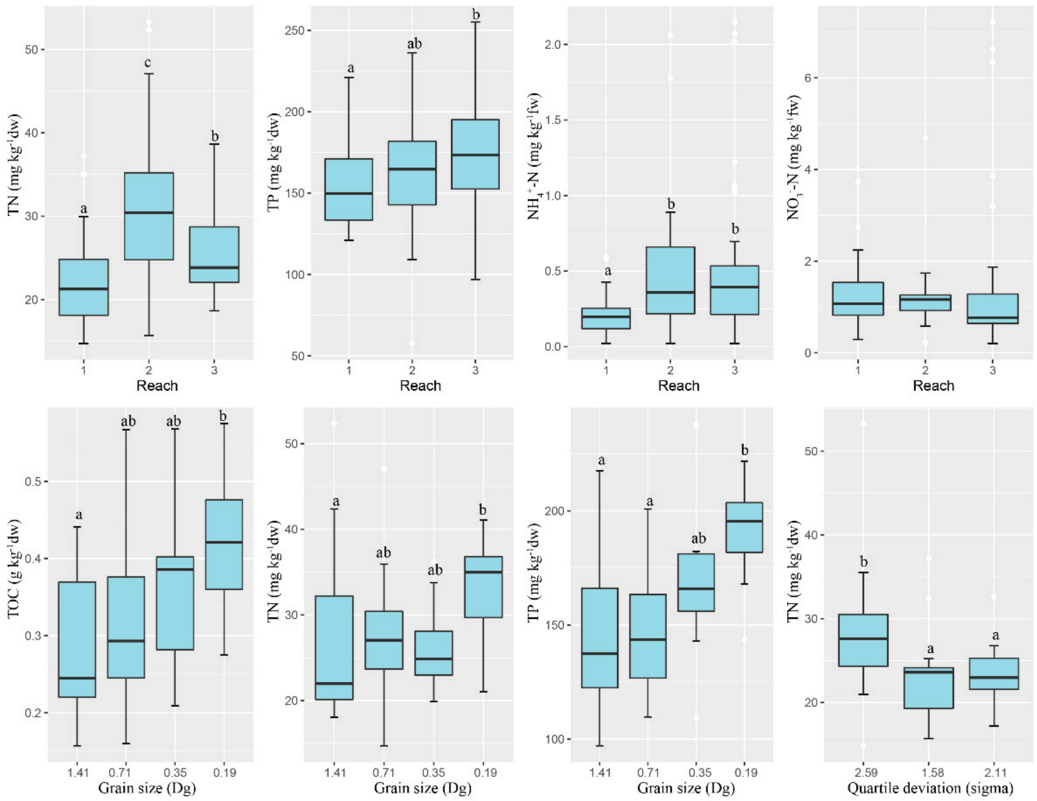

Figure S4. The chemical parameters of the sandy sediment with different reaches, particle median sizes, and heterogeneities. Different letters indicate significant differences ($p < 0.05$).

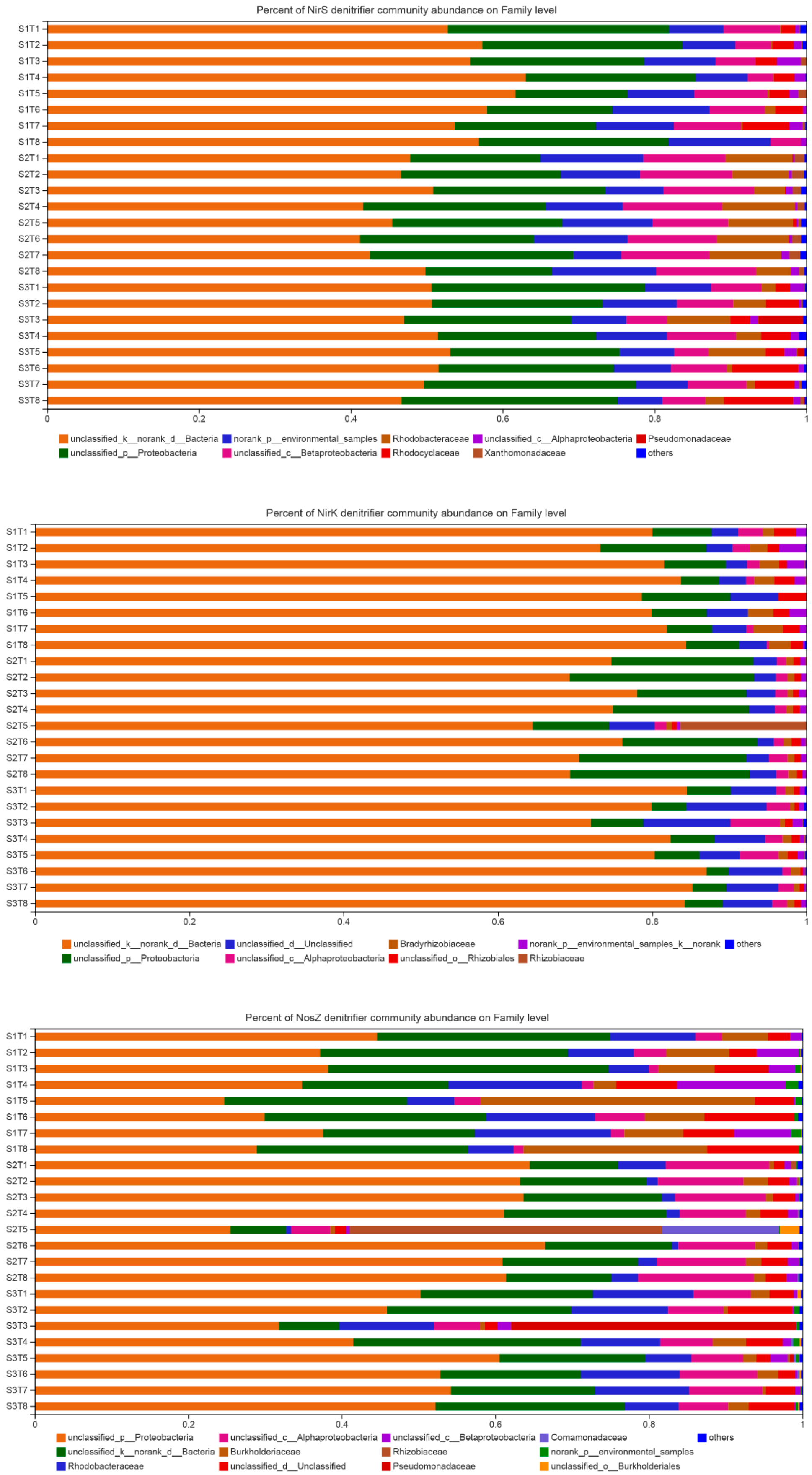

Figure S5. Three denitrifier community compositions (at the family level) within 24 different treatments.
